# Novel Missense Variants of ZFPM2/FOG2 Identified in Conotruncal Heart Defect Patients Do Not Impair Interaction with GATA4

**DOI:** 10.1371/journal.pone.0102379

**Published:** 2014-07-15

**Authors:** Wenwen Zhang, Li Shen, Zhantao Deng, Yibing Ding, Xuming Mo, Zhengfeng Xu, Qian Gao, Long Yi

**Affiliations:** 1 Center for Translational Medicine, Nanjing University Medical School, Nanjing, Jiangsu, PR China; 2 Jiangsu Key Laboratory for Molecular Medicine, Nanjing University Medical School, Nanjing, PR China; 3 Department of Cardiothoracic Surgery, Shanghai Children's Hospital, Shanghai Jiaotong University, Shanghai, PR China; 4 Department of Cardiothoracic Surgery, Nanjing Children's Hospital, Nanjing, PR China; 5 Center of Prenatal Diagnosis, Nanjing Maternity and Child Health Hospital, Nanjing Medical School, Nanjing, Jiangsu, PR China; New York Medical College, United States of America

## Abstract

Conotruncal heart defect (CTD) is a complex form of congenital heart disease and usually has a poor prognosis. *ZFPM2/FOG2* encodes a transcription cofactor that interacts with GATA4 to regulate cardiac development. This regulation has been established in knockout mouse models that display a range of heart malformations, especially CTD. Although previous studies have identified several missense variants in *ZFPM2/FOG2* that may cause CTD, it remains unclear whether they are involved in CTD pathogenesis because the study populations were limited and the functional status was unknown. In this report, we screened a larger CTD population, which comprised 145 tetralogy of Fallot (TOF), 37 double-outlet ventricle outflow (DORV), and 18 transposition of the great artery (TGA), to investigate exon mutations as well as copy number variations in *ZFPM2/FOG2*. Four variants (p.V339I in one DORV, p.A426T in one DORV, p.M703L in three TOF, p.T843M in one TOF) were found in six patients, of which two are reported here for the first time. No copy number variations of the gene were detected. GST pull-down assays demonstrated that all potentially deleterious variants, including those previously reported, did not impair the interaction with GATA4, except for variant p.M544I and p.K737E, which subtly impaired the binding. Thus, these missense variants may be involved in other mechanisms underlying CTD or may be unrelated to CTD occurrence.

## Introduction

Conotruncal heart defect (CTD), a subtype of congenital heart disease (CHD), is estimated to occur in approximately 1 per 1000 live births [1]. CTD, comprising primarily the defects tetralogy of Fallot (TOF),transposition of the great artery (TGA), and double-outlet ventricle outflow (DORV), is a complex form of CHD that requires surgical repair once diagnosed. Although the etiology of the majority of CTD remains obscure, several studies have implicated genetic factors in its pathogenesis. In fact, 22q11 microdeletion, responsible for DiGeorge syndrome, accounts for roughly 12% of conotruncal anomalies [2].

CTD stems from perturbation of outflow tract (OFT) morphogenesis. A recently identified second heart field (SHF), which gives rise to OFT, may provide insights into the mechanism underlying CTD [3]. Indeed, SHF is subject to delicate regulation by various transcription factors and cofactors such as GATA4 and NKX2-5. Consistent with this finding, multiple mutations in the genes encoding these factors have been identified in CTD patients [4], [5].

ZFPM2/FOG2, a transcription cofactor, forms a complex with GATA4 and plays a crucial role in heart development, especially OFT [6]. Accordingly, *ZFPM2/FOG2* knockout mice recapitulate the abnormal heart phenotype, including CTD [7], [8]. Moreover, mice harboring a GATA4 mutation that disrupts its interaction with ZFPM2/FOG2 have features similar to *ZFPM2/FOG2* null embryos [9]. Pizzuti et al. first sequenced *ZFPM2/FOG2* among 47 TOF patients and revealed two mutations, whereas the results of functional analyses implied that they had no or only mild effects on gene function [10]. Subsequent independent studies also identified several mutations, mainly in DORV, but did not further investigate their pathogenicity [11], [12]. With the advent and application of next-generation sequencing, large-population sequencing projects, such as the Exome Sequencing Project (ESP) and ClinSeq, have revealed in normal participants many variants that were previously assumed to be deleterious [13]. These findings thus highlight the necessity to discern pathological mutations from benign polymorphisms to unravel the precise molecular mechanisms underlying disease. Besides mutations, recent studies have revealed copy number variations (CNVs) that may be involved in CTD development [14]. Indeed, *ZFPM2/FOG2* is located in a chromosomal region that is prone to break, and thus CNVs of the gene could cause CTD by altering gene dosage [15].

Although ZFPM2/FOG2 mutations have been found in CTD patients, their functional significance is not known and the study populations and mutation spectra have thus far been limited. To further explore the role of *ZFPM2/FOG2* in CTD, we therefore screened for mutations and CNVs in a larger CTD cohort and assessed potentially deleterious mutants with respect to their ability to bind GATA4.

## Materials and Methods

### Subjects

From January 2004 to January 2006, blood samples were collected from CTD patients who were admitted to Nanjing Children's Hospital in Jiangsu Province and Shanghai Children's Hospital in Shanghai. The diagnoses were made by experienced physicians on the basis of echocardiography and were validated by subsequent surgery. Patients with major extracardiac abnormalities were excluded. Genomic DNA was extracted from peripheral blood leukocytes using standard procedures (Qiagen). DNA was then subjected to 22q11 microdeletion analysis [16]. Only patients without such cytogenetic aberrations were included for further research, which comprised 145 TOF, 37 DORV, and 18 TGA

As a control group, 100 unrelated healthy individuals were recruited at the Center of Physical Examination of Nanjing Children's Hospital. The control group had no history of CHD, and echocardiography revealed no heart structure lesions.

### Ethics Statement

The study was approved by the Ethics Committee of Nanjing University Medical School, Nanjing Children's Hospital, and Shanghai Children's Hospital. Informed written consent was obtained from children's parents or guardians and from controls.

### DNA Sequencing and Bioinformatics Analysis

Human *ZFPM2/FOG2* is located on 8q23.1 and is encoded by eight exons. All exons and splice sites of *ZFPM2/FOG2* were amplified by PCR using 14 pairs of *ZFPM2/FOG2* gene-specific primers designed by the online software Primer 3, of which 7 pairs were directed to the long sequence of exon 8 (Table S1). PCR fragments were purified with the Gel Extraction kit (Omega), subjected to cycle sequencing using the BigDye Terminator v3.1 kit (Applied Biosystems), and injected into the ABI 3100 Genetic Analyzer (Applied Biosystems).

Sites of variation were identified by referring to the ZFPM2/FOG2 sequence derived from GenBank (accession number: NM_012082.3). Altered nucleotides were confirmed by sequencing both strands. The novelty of all variants was determined from the National Center for Biotechnology Information (NCBI) human SNP database (dbSNP), the 1000 Genomes Project database, and the ESP. The protein sequences of other species were obtained from GenBank, and conservation analysis was performed with Clustalw2 software. Any missense variants identified were subjected to the bioinformatics tool PolyPhen-2 to evaluate the possible effects on protein structure and function.

### MLPA (Multiplex Ligation-dependent Probe Amplification) Synthetic Probe Design and Assay

The target sequence of each synthetic half-probe was designed according to the guidelines provided by MRC-Holland. For the *ZFPM2/FOG2* probe set (Table S2), one probe pair was designed for each of the eight exons of *ZFPM2/FOG2*. Corresponding oligonucleotides were purchased from Generay Biotech. The P300-A1 Human DNA Reference-2 kit (MRC-Holland) was employed to provide reference probes and control fragments specific for unique human DNA sequences. Synthetic MLPA probes were added to the probe mix provided by the kit. The MLPA assay was performed according to the standard procedure [17]. Data were analyzed using Gene Mapper software (Applied Biosystems), and relative signal values were calculated. Threshold values for deletion and duplication were set at 0.7 and 1.3, respectively.

### Analysis of Protein–Protein Interactions

Human *ZFPM2/FOG2* cDNA was obtained from Generay Biotech, Shanghai. Mutated cDNA was acquired by PCR-mediated mutagenesis with the appropriate primers. DNA sequencing confirmed the wild-type and mutant forms, which were then cloned into vector PGEX-4T-1 and expressed under isopropyl β-D-1-thiogalactopyranoside induction. The N-terminal zinc-finger sequence of GATA4 (residues 200–254) was inserted into vector pSUMO-Mut and expressed under IPTG induction. Interactions between ZFPM2/FOG2 (wild type and variants) and GATA4 were examined by GST-mediated pull-down assays using the Pierce GST Protein Interaction Pull-Down kit (Thermo Scientific). Wild-type or mutant ZFPM2/FOG2 was bound to 200 µl of glutathione resin as a GST-fusion protein and incubated with GATA4 fragment at 4°C. After extensive washing with a buffer containing 25 mM Tris•HCl (pH 7.2) and 150 mM NaCl, the complex was eluted with elution buffer supplied in the kit and visualized by SDS-PAGE and Coomassie staining. Image analysis was performed to determine the band intensities by using Photoshop tools.

## Results

**Figure 1:**
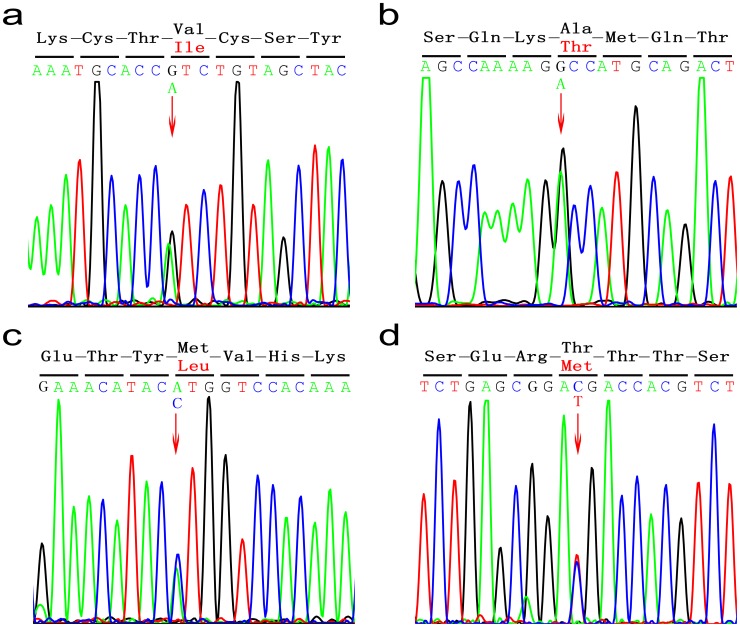
Sequencing chromatograms of four heterozygous missense variants. (A) Each arrow indicates the position of the nucleotide variant that results in the substitution of wild-type Val with Ile at codon 339. (B) Substitution of wild-type Ala with Thr at codon 426. (C) Substitution of wild-type Met with Leu at codon 703. (D) Substitution of wild-type Thr with Met at codon 843.

**Figure 2:**
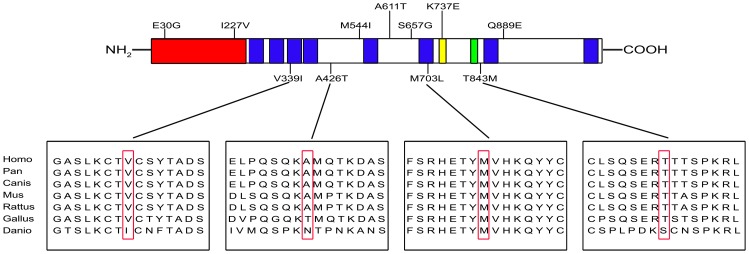
Locations of mutations identified and sequence alignments in ZFPM2/FOG2. Top, the N-terminal transcriptional repression domain is indicated in red; the eight zinc-finger motifs are represented by blue; the nuclear localization signal is indicated in yellow; the putative CtBP-binding site is represented by green. Variants identified in this study are shown underneath the protein structure; variants reported in previous studies are shown above. Bottom, multiple alignment of partial amino acid sequences of human ZFPM2/FOG2 and its homologs from other species. Variant residues are boxed. Accession numbers of the sequences used are as follows: Homo, NP_036214.2; Pan, XP_001158075.1; Canis, XP_539118.2; Mus, NP_035896.1; Rattus, XP_235253.4; Gallus, XP_418380.2; Danio, NP_001034724.1.

Four *ZFPM2/FOG2* heterozygous variants were identified in six CTD patients (p.V339I in DORV C121, p.A426T in DORV C21, p.M703L in TOF C067, C079,C183, p.T843M in TOF C076) (Figure 1). These mutations were absent in 100 healthy control individuals of the same ethnic background, suggesting that they might cause disease. Variants p.V339I and p.M703L had been reported previously, whereas p.A426T and p.T843M were new variants found in our CTD cohort. Variants p.V339I, p.A426T, and p.M703L were found in the dbSNP and 1000 Genomes Project databases with the following rs IDs: rs201845067, rs35843564, and rs121908603. Moreover, variants p.V339I and p.A426T were also found in the ESP database. Variant p.T843M, however, was not identified in any of these databases (Table 1). Notably, all four variants had variants at positions that were highly conserved throughout species; especially, Met 703 was conserved in all species including zebrafish (Figure 2).

**Table 1:** *ZFPM2* missense variants identified in this and previous studies.

Nucleotide substitution	Amino acid change	CTD type, (patient number)	Report status	Database information	Protein domain	PolyPhen -2 prediction	Inheritance status
c. 1015G>A^12^	p.V339I	DORV(1)/DORV(1)	This study/previously reported	rs201845067/1000 Genomes with MAF 0.001/ESP	Zinc-finger motif 1	Benign	NA/De novo
c. 1276G>A	p.A426T	DORV(1)	This study	rs35843564/1000 Genomes with MAF 0.005/ESP	Unknown	Benign	NA
c.2107A>C^12^	p.M703L	TOF(3)/TOF(1)	This study/previously reported	rs121908603/1000 Genomes with MAF 0.001	Zinc-finger motif 6	Possibly damaging	Inherited/De novo
c. 2528C>T	p.T843M	TOF(1)	This study	none	Unknown	Probably damaging	NA
c. 89A>G^10,11^	p.E30G	TOF(1)/DORV(1)	Previously reported	rs121908601/1000 Genomes with MAF:0.002/ESP	N-terminal transcriptional repression domain	Possibly damaging	Inherited/NA
c. 679A>G^11^	p.I227V	DORV(1)	Previously reported	rs202204708/ESP	N-terminal transcriptional repression domain	Probably damaging	NA
c. 1632G>A^11^	p.M544I	TOF(1)	Previously reported	rs187043152/1000 Genomes with MAF 0.002/ESP	Unknown	Benign	NA
c. 1831G>A^12^	p.A611T	TOF(1)	Previously reported	none	Unknown	Benign	De novo
c. 1968A>G^10^	p.S657G	TOF(1)	Previously reported	rs28374544/1000 Genomes with MAF 0.033/ESP	Unknown	Benign	Inherited
c. 2209A>G^12^	p.K737E	DORV(1)	Previously reported	none	Nuclear localization signal	Probably damaging	NA
c. 2665C>G^12^	p.Q889E	DORV(1)	Previously reported	rs146423225/1000 Genomes vwith MAF 0.006/ESP	Unknown	Benign	De novo

CTD, conotruncal heart defect; DORV, double-outlet ventricle outflow; MAF, minor allele frequency; ESP, Exome Sequencing Project; NA, not available.

ZFPM2/FOG2 is a transcription cofactor that contains eight zinc-finger motifs that are crucial for its function. Of the variants found in this study, two (p.V339I and p.M703L) contained mutations that were located in zinc-finger regions, suggesting that their function might be disrupted (Figure 2). Furthermore, using PolyPhen-2 in silico analysis to evaluate their potential pathogenesis [18], variants p.V339I and p.A426T were predicted to be benign, whereas variants p.M703L and p.T843M were considered to be deleterious. Finally, only the parents of patient C067 and C183 bearing p.M703L were available for study; these individuals had no history of CHD, and no cardiac abnormities were observed by echocardiography. Sequencing their genomic DNA revealed that one of the parents has an identical nucleotide substitution, suggesting that it is heritable (Table 1).

**Figure 3:**
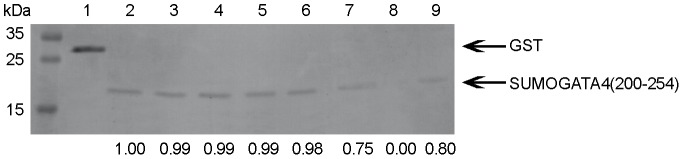
Interactions between Zfpm2 variants and Gata4 assessed by GST pull-down assays. Coomassie blue staining indicated the amounts of GST fusions pulled down in the assays. The relatively band intensities compared with the wild type are shown in the bottom. Molecular mass standards (kDa) are indicated at the left. Lane 1, GST; Lane 2, GST-Zfpm2 wild type; Lane 3, GST-Zfpm2 p.M703L; Lane 4, GST-Zfpm2 T843M; Lane 5, GST-Zfpm2 E30G; Lane 6, GST-Zfpm2 I227V; Lane 7, GST-Zfpm2 K737E; Lane 8, GST-Zfpm2 S402R; Lane 9, GST-Zfpm2 M544I.

MLPA analysis revealed that normalized MLPA values were within the normal range for all exons in all subjects, thereby excluding a major contribution of *ZFPM2/FOG2* CNVs in the pathogenesis of the investigated CTDs. To date, 11 variants have been identified in CTD patients, although their roles remain unclear (Table 1). ZFPM2/FOG2 reportedly regulates cardiac-specific gene expression via formation of a complex with GATA4. To determine whether the missense mutations disrupt the interaction between ZFPM2/FOG2 and GATA4, we performed GST pull-down assays. We tested the five variants (p.E30G, p.I227V, p.M703L, p.K737E, and p.T843M) that were predicted by PolyPhen-2 to be deleterious to protein function. Two another variants (p.M544I and p. S402R) were also included given its positive findings in binding assays demonstrated in a recent study [19]. The results showed that wild-type ZFPM2/FOG2 pulled down GATA4 specifically. Consistent with the previous study, variant p.S402R, yet not identified in CTD patients, abolished the interaction and variant p. M544I reduced the interaction, whereas the degree of change differed (Figure 3). The binding capacities with GATA4 remained intact in four variants (p.E30G, p.I227V, p.M703L, and p.T843M) except variant p. K737E subtly impaired the binding. Those results suggest that variants identified in CTD patients might not contribute to disease development, at least not by abolishing the formation of the ZFPM2/FOG2–GATA4 complex.

## Discussion

CHD is a common disease with a varied genetic background. Mutations in genes that encode factors involved in cardiac morphogenesis and development could lead to heart malformations. These factors are mainly categorized into three groups: transcription factors, signal molecules, and structural proteins [20]. ZFPM2/FOG2 is an important transcriptional regulator of cardiogenesis. Its role has been verified in knockout mice that exhibit a variety of cardiac defects, including septal, valve, and outflow tract anomalies [8]. In agreement with these observations, ZFPM2/FOG2 variants have been reported in TOF and DORV patients, whereas they are absent in normal controls [10]–[12], [21]. Because of the genetic complexity of CTD, it is unknown whether the variants identified in CTD cohorts are causative. First, all variants found result from missense mutations that need to be functionally characterized; frameshift mutations caused by insertion or deletion, nonsense mutations, and intragenic CNVs that have detrimental effects on protein function have not been identified. Second, ZFPM2/FOG2 mutation pedigree reports for co-segregation analysis are not available. Finally, many missense mutations are included in the dbSNP and 1000 Genomes Project databases, and some of them are passed on from parents with no cardiac anomalies, suggesting that they are shared among patients and the healthy subjects.

Distinguishing deleterious mutations from those that are benign is a crucial step toward deciphering their role in CTD development. The cost of next-generation sequencing continues to decrease, and this has enabled production to large amounts of whole-genome data. Surprisingly, many missense mutations that were previously considered to cause a specific disease are also present in healthy people, emphasizing the need to confirm their mechanistic roles [22]. ZFPM2/FOG2 forms a complex with GATA4 to regulate downstream gene expression rather than directly promoting transcription. Mice that express GATA4 with a single amino acid replacement that abrogates its interaction with ZFPM2/FOG recapitulate the ZFPM2/FOG2 knockout phenotype, suggesting that ZFPM2/FOG2 function relies on GATA4 binding [9]. Of three studies that screened CTD populations for ZFPM2/FOG2 mutations, only one further explored their effects on function; variants p.E30G and p.S657G have no impact on the ZFPM2/FOG2 interaction with GATA4, and they have undetectable or minimal effects on downstream gene expression [10]. We conducted a larger CTD population screen, identified four variants, and investigated the GATA4 binding of all of the potentially deleterious ZFPM2/FOG2 mutations reported so far. Our findings agreed with a previous study reporting that missense variants identified in CTD patients exert mild effects, if any, on the ZFPM2/FOG2–GATA4 interaction. It has been shown that a ZFPM2/FOG2 fragment containing only zinc fingers 5 and 6 is sufficient to interact with GATA4 [23]. Given that the majority of the mutations tested lie beyond this domain, our results are not surprising. Notably, variant p.M703L is postulated to be functionally impaired because this mutation lies within zinc finger 6 and has been identified multiple times in CTD patients. Moreover, Met 703 is conserved among all species, and substitution to Leu is predicted by PolyPhen-2 to be deleterious to the protein function. In our study, however, the results of GST pull-down assays did not support our prediction.

Our study is limited by exploring only several variants rather than all. The selection of variants was based on the in silico results of PolyPhen-2, which has been reported to have 73% sensitivity and 70% specificity [24]. Thus, our results may in fact be representative of CTD patients in general. The issue of whether missense variants found in CTD patients are causative remains unresolved, however, because we have not investigated all aspects of ZFPM2/FOG2 function. For example, variant K737E lies in the nuclear localization signal sequence and thus may interrupt its nuclear entry [25]. Furthermore, given that the interaction of certain variants with GATA4 remains intact, variants that have mutations in the N-terminal transcriptional repression domain of ZFPM2/FOG2 (p.E30G and p.I227V) may influence downstream gene expression. However, it has been reported that variant p.E30G does not repress GATA4-mediated gene expression in the luciferase reporter assay [10]. To fully investigate these variants, it is necessary to identify the downstream genes involved in cardiac development and assess the effects of these variants on transcription of the downstream genes.

In conclusion, we expanded the ZFPM2/FOG2 variant profile in CTD patients by discovering two new missense variants (p.A426T and p.T843M). No large intragenic deletions or duplications of this gene were found. The results of GST pull-down assays revealed that the missense mutations most likely to cause structural damage had no mild effect, if any, on the interaction with GATA4. Thus, the effects of these mutations may involve other known or unknown mechanisms underlying CTD or, alternatively, the mutations identified may not cause CTD. Our findings are a reminder for physicians to carefully evaluate gene variants associated with certain diseases, especially those lacking robust functional evidence, to confirm their pathogenicity.

## Supporting Information

Table S1 Primers used for PCR and sequencing of ZFPM2 exons.
(DOCX)

Table S2 Synthetic probes corresponding to ZFPM2 eight exons utilized in MLPA assays.
(DOCX)
