# Supplementary material for: Novel Missense Variants of ZFPM2/FOG2 Identified in Conotruncal Heart Defect Patients Do Not Impair Interaction with GATA4
Source: PLoS One. 2014 Jul 15;9(7):e102379. doi: 10.1371/journal.pone.0102379 (PMC4099368; doi:10.1371/journal.pone.0102379)
Supplement: Table S1 — Primers used for PCR and sequencing of ZFPM2 exons. (DOCX) [file pone.0102379.s001.docx]

| Supplementary Table 1. Primers used for PCR and sequencing | | | |
| --- | --- | --- | --- |
| Exon | Primer | Sequence* | Amplicon size |
| 1 | Forward | TCTCTTCTCTCATTTGCTTGCTC | 287 |
|  | Reverse | GGCAATAATCCCACCAACTCC |  |
| 2 | Forward | TGTCTTCCTTGCATATTTTTGGT | 299 |
|  | Reverse | GTCAGCACTCTGAGGACTATTATG |  |
| 3 | Forward | TGATAAGGACATCCCTTTATGAGG | 394 |
|  | Reverse | CAAGCAGAGGTAGCACTTTGG |  |
| 4 | Forward | AGGTGGCTGCTGATAAAGTACAA | 240 |
|  | Reverse | GCTATGGCAAAATACTAACAGTCG |  |
| 5 | Forward | GGTTTGGGAGATTTAGTTGTTTGT | 284 |
|  | Reverse | CTGTTTTGCAATGCTCAGGTT |  |
| 6 | Forward | CAGACTCAAGCATCCTATGTCAA | 310 |
|  | Reverse | ATAAATTCCCATCACCTCTAAAGC |  |
| 7 | Forward | GTTTCAAATGGACAGCAGCAA | 319 |
|  | Reverse | TAACAGAGTCTTCTGGAGCAACA |  |
| 8-1 | Forward | TTAGAAAAGGTCCCTGTCATTCA | 363 |
|  | Reverse | GCTTCTGGTCAATAAGTCTGTGG |  |
| 8-2 | Forward | CAAGTGCAACTGAAGACAGCTTAC | 403 |
|  | Reverse | TAGCTACTACTGCCATGCCTCA |  |
| 8-3 | Forward | CCTTTCCTATCTCAGTTTTCTTTCC | 481 |
|  | Forward | AGGTGGAGAGCTTCTTAGTTTGAG |  |
| 8-4 | Forward | CTTCAAACATCTTGCATCAATTCT | 429 |
|  | Reverse | TACGTCAAGAAATCTCTGCTGAAC |  |
| 8-5 | Forward | CGCAGAAAGATGTATGAGATGTG | 408 |
|  | Reverse | CTTGTGGGCCAGATAGTTTTCTA |  |
| 8-6 | Forward | CTTGTGGGCCAGATAGTTTTCTA | 471 |
|  | Reverse | CGCAGAAAGATGTATGAGATGTG |  |
| 8-7 | Forward | GCACTGTGTGCAAGATCAGTTT | 495 |
|  | Reverse | GCACTGTGTGCAAGATCAGTTT |  |
| *primer sequence nucleotides are from 5' to 3' | | |  |
