# Supplementary material for: Novel Missense Variants of ZFPM2/FOG2 Identified in Conotruncal Heart Defect Patients Do Not Impair Interaction with GATA4
Source: PLoS One. 2014 Jul 15;9(7):e102379. doi: 10.1371/journal.pone.0102379 (PMC4099368; doi:10.1371/journal.pone.0102379)
Supplement: Table S2 — Synthetic probes corresponding to ZFPM2 eight exons utilized in MLPA assays. (DOCX) [file pone.0102379.s002.docx]

| Supplementary Table 2. Synthetic probes corresponding to ZFPM2 eight exons | | |  |
| --- | --- | --- | --- |
| Probe name | 5' half hybridization sequence^a^ | 3' half hybridization sequence^b^ | Size^c^ |
| ZFPM2-ex3 | GATGAAGGAATCCAGGAGACAGCAGAATC | AGATGGGGACACACAGTCAGAGAAACCGG | *100* |
| ZFPM2-ex5 | CAATGGTGCTGACTGCTGGTCCCAAGTGGT | TGCTGGATGTGACTTGGCAAGGAGTGGAAG | *102* |
| ZFPM2-e4 | TTCAGAGTCGACAGCAACTTCCAGTGGGAACAACC | TGGGGGCCGTTTCCTGGGAAGATGGACTTGAATAA | *112* |
| ZFPM2-e2 | GGGCCGCTTGAAGATGCCATTGAAGATGAGGAAGAAGA | ATGTCCATCAGAGGAAACAGACATCATCTCCAAAGGAG | *118* |
| ZFPM2-e1 | CTCCTCACTGTCACACTCTCTGTGCCCCCGTCTCTCTTCT | CTCATTTGCTTGCTCATCTCCGAACGTGAATCCGCGGCTC | *122* |
| ZFPM2-e6 | GGTCAGCTTTGGTGTACAACTACGAAGGCCATCTCTGAGGGTGAA | GAGCTAATTGCCTTTGTGGTGGATTTTGACTCAAGGCTACAAGCT | *132* |
| ZFPM2-e7 | CATCTGGTATCGGAGTGAGCGGAATCTGCAGGCCCATTTGATGTACTA | CTGCAGTGGGAGGCAAAGAGAAGCTGCTCCGGTGTCAGAGGAAAATGA | *138* |
| ZFPM2-e8 | GTTACTGCACATCAGCGTAATGACCTGGGTCAACTGGACGGCAAAGTGTT | TCCGAATCCAGAAAGCGAACGAAACAGCCCTGATGTCAGCTACGAAAGAA | *142* |
| ^a^ The 5' half-probes are preceded by the universal tag sequence GGGTTCCCTAAGGGTTGGA | | |  |
| ^b^ The 3' half-probes are followed by the universal tag sequence CTAGATTGGATCTTGCTGGCAC and are phosphorylated at the 5' end | | |  |
| ^c^ Size of the ligation product in bp | | |  |
